# Impact of *In* *vitro* Gastrointestinal Digestion on the Chemical Composition and Prebiotic Potential of Coffee Silverskin

**DOI:** 10.1007/s11130-025-01390-z

**Published:** 2025-09-05

**Authors:** Marlene Machado, Iva Fernandes, Ana Fernandes, Liliana Espírito Santo, Cláudia Passos, Aroa Santamarina, Alejandra Cardelle-Cobas, Manuel A. Coimbra, Maria B. P. P. Oliveira, Helena Ferreira, Rita C. Alves

**Affiliations:** 1 REQUIMTE/LAQV, Department of Chemical Sciences, Faculty of Pharmacy, University of Porto, Rua Jorge Viterbo Ferreira, 228, 4050-313 Porto, Portugal; 2 Department of Chemistry and Biochemistry, Faculty of Sciences, University of Porto, Rua do Campo Alegre, 4169-007 Porto, Portugal; 3 REQUIMTE/LAQV, Department of Chemistry and Biochemistry, Faculty of Sciences,, University of Porto, Rua do Campo Alegre, 1021-1055, 4169-007 Porto, Portugal; 4 REQUIMTE/LAQV, Department of Chemistry, Campus Universitário de Santiago, University of Aveiro, 3810-193 Aveiro, Portugal; 5 Laboratory - Food Hygiene, Inspection and Control, Department of Analytical Chemistry, Nutrition and Bromatology, University of Santiago de Compostela, 27002 Lugo, Spain; 6 i4HB, UCIBIO - Research Unit on Applied Molecular Biosciences, Department of Biological Sciences, Faculty of Pharmacy, University of Porto, Rua Jorge Viterbo Ferreira, 228, 4050-313 Porto, Portugal

**Keywords:** Coffee by-product, Probiotic, Organic acids, Antioxidant activity

## Abstract

The growing interest in prebiotic ingredients has led to the valorization of agri-food by-products, such as coffee silverskin, known for its richness in dietary fiber and health-promoting compounds. This study evaluated the impact of *in vitro* simulation of gastrointestinal digestion on the chemical composition (carbohydrates, caffeine, and chlorogenic acids) and prebiotic potential (probiotic growth, organic acid production, pH, and antioxidant activity) of milled coffee silverskin. The results show stability of polysaccharides during digestion, while caffeine and 5-caffeoylquinic acid were partially released into the bioaccessible fraction. The incubation of digested sample (DS) and the control (CS) with *Lacticaseibacillus paracasei* subsp. *paracasei* and *Lactiplantibacillus plantarum* subsp. *plantarum* resulted in probiotic growth, an increase in acetic acid production, and pH decrease. Following incubation, the cell-free supernatants of the DS and CS exhibited greater antioxidant activity compared to the negative control, glucose, and fructooligosaccharides. This study shows the functional potential of the whole silverskin due to its prebiotic and antioxidant properties.

**Supplementary Information:**

The online version contains supplementary material available at 10.1007/s11130-025-01390-z.

## Introduction

Silverskin, a film that detaches from green coffee beans during roasting, represents approximately 4% of the green coffee beans, with an annual production of 7,500 tons of silverskin, which is generally discarded as solid waste [1, 2]. This by-product is a rich source of several components including dietary fiber (57–66%), protein (15–21%), ash (9%), lipids (2–3%), melanoidins, caffeine and polyphenols (mainly chlorogenic acids) [3–5]. Silverskin has been previously explored as a functional ingredient with prebiotic and antioxidant properties due to its richness in dietary fiber and chlorogenic acids [6]. Prebiotics exhibit a range of positive biological actions for human health, including modulation of gut microbiota, of the gut-brain axis, promotion of cardiovascular and bone health, modulation of the immune system, and prevention of metabolic diseases [7, 8]. The first requirement for an ingredient to be considered prebiotic is to be resistant to gastrointestinal digestion (GID) (gastric juice, enzymatic hydrolysis, and gastrointestinal absorption). It must then reach the colon, be metabolized by the resident microbiota and selectively stimulate the activity or growth of beneficial bacteria [6]. Although studies on the prebiotic potential of silverskin are still limited, some studies have reported promising results. Borrelli et al. [9] and Jiménez-Zamora et al. [10] found that silverskin, after faecal fermentation, can promote the growth of probiotic strains such as bifidobacteria and lactobacillus. More recent studies have found similar effects with silverskin extracts rich in soluble fiber and bioactive compounds [11, 12] or silverskin xylooligosaccharides [13] including the production of organic acids and a decrease in pH, suggesting a possible functional action in the colon.

Despite these findings, relevant shortcomings remain. No study has clearly delineated the effects of *in vitro* GID on the chemical composition of silverskin, especially the carbohydrate fraction and the bioactive profile, both of which influence its prebiotic activity. Evaluating the chemical and structural stability of prebiotics during their passage through the upper gastrointestinal tract is critical because alterations caused by this environment can alter functionality and, subsequently, interaction with the colonic microbiota [14]. As far as we know, the impact of *in vitro* GID has only been studied in silverskin extracts in terms of bioactive compounds [15, 16]. Moreover, although there have been studies on the prebiotic activity of silverskin employing faecal microbiota, research on specific probiotic strains is limited. Finally, it remains to be observed whether the probiotic fermentation of silverskin can enhance other functional properties, such as antioxidant activity, that are relevant to gut health.

Therefore, this study aimed to evaluate the impact of *in vitro* GID on chemical composition and prebiotic potential of coffee silverskin compared to a control obtained by aqueous extraction. The following parameters were investigated for both digested and control silverskin: carbohydrates profile; caffeine content and chlorogenic acids profile; effect on the growth of probiotic strains (*Lacticaseibacillus paracasei* subsp. *paracasei* BAA-52 ATCC and *Lactiplantibacillus plantarum* subsp. *plantarum* NCTC 13644), production of organic acids, pH change, and antioxidant activity.

## Materials and Methods

(See Supplementary Section)

## Results and Discussion

GID is a complex and dynamic process that aims to make nutrients and bioactive compounds available for intestinal absorption. This process involves physiological factors such as temperature, pH, digestive enzymes and colonic bacteria, which influence the physicochemical properties of nutritional substances [17, 18]. Since resistance to upper GID is a key criteria for prebiotics, Sect. "Carbohydrates Content and Composition" and "Composition of Caffeine and Chlorogenic Acids" focus on the non-bioaccessible fractions (CS and DS) that reach the colon, analysing their carbohydrate composition, caffeine, and caffeoylquinic acids.

### Carbohydrates Content and Composition

Table 1 shows the monosaccharide composition (mol%) of coffee silverskin comparing the control (CS) and the digested silverskin (DS). Both CS and DS exhibited a carbohydrate content of 30%, with a similar component sugars composition, rich in uronic acids (36 and 33 mol%, respectively), xylose (22 and 18 mol%), glucose (20 vs. 18 mol%), and containing still galactose (7 and 12 mol%), fucose (3 and 8 mol%), arabinose (9 and 6 mol%), mannose (3 and 5 mol%),and rhamnose (1 mol%). A significant increase in fucose (mol%) was observed in DS, which may result from a relative, non-significant loss of other monosaccharides during digestion, rather than an absolute increase in fucose content. The high content of uronic acids and the presence of galactose and arabinose in silverskin indicates that pectic polysaccharides are a predominant structural element in silverskin, in accordance with literature [19]. The high glucose content is characteristic of cellulose, although glucose content, together with xylose, galactose, and fucose suggests the presence of branched xyloglucans, commonly found in primary cell walls of dicotyledonous species [20].

**Table 1:** Carbohydrate composition of control and digested coffee silverskin

Carbohydrate composition (mol%)	Control silverskin (CS)	Digested silverskin (DS)
Rha	1.4 ± 0.6	0.8 ± 0.1
Fuc	2.5 ± 0.1	7.5 ± 1.5*
Ara	8.5 ± 0.1	6.2 ± 2.6
Xyl	21.7 ± 0.3	18.0 ± 2.1
Man	3.0 ± 0.0	4.9 ± 1.8
Gal	6.6 ± 0.4	12.0 ± 5.1
Glc	20.1 ± 0.6	18.0 ± 1.8
UA	36.1 ± 2.1	32.5 ± 1.2
Total Carbohydrates (g/kg)	302.8 ± 22.2	296.2 ± 29.9

Results represent the average of 3 independent experiments ± SD. Sugar residues: Rha, rhamnose; Fuc, Fucose; Ara, arabinose; Xyl, Xylose; Man, mannose; Gal, galactose; Glc, glucose; UA, uronic acids. *, significant differences between CS and DS at *p* < 0.05

The data suggest that *in vitro* GID did not influence the primary structure of the silverskin polysaccharides. Free sugars accounted for 2% of the carbohydrates, which is in agreement with the negligible variation of carbohydrate content after digestion. These results showed that silverskin carbohydrates are resistant to digestion and absorption in the small intestine, allowing them to enter the colon and possibly interact with the local microbiota to stimulate the production of short-chain fatty acids (SCFA).

### Composition of Caffeine and Chlorogenic Acids

Compared to the carbohydrate fraction, the bioactive compounds analysed were less stable throughout *in vitro* GID. Indeed, digestion promoted the release of caffeine and 5-caffeoylquinic acid into the intestinal fluid. Nonetheless, a significant amount of caffeine (5.41 and 3.65 mg/g dw in CS and DS, respectively) can still reach the colon and influence the composition of the gut microbiota. 5-CQA concentrations were 0.15 mg/g in the CS and 0.09 mg/g in the DS, indicating significant preservation of the compound during *in vitro* GID. This decrease may be related to pH variations during GID. 5-CQA is stable at pH 3 to 4, but in strongly acidic and alkaline conditions it can undergo isomerisation and hydrolysis [21]. These results are consistent with the Literature, which suggests that approximately two-thirds of ingested 5-CQA reaches the colon intact and can benefit the gut microbiota [22]. 5-CQA has been linked with promoting the growth of *Lactobacillus* and *Bifidobacterium* [23, 24] as well as positively modulating the gut microbiota even in disease conditions such as colitis [25] and alcoholic liver disease [26].

In this study, the 3-CQA and 4-CQA isomers were not detected in either the DS or the CS, likely due to their solubilisation into the bioaccessible fraction (supernatant, not analysed) during *in vitro* digestion or aqueous extraction.

### Screening Prebiotic Effect

#### Evaluation of Silverskin on Probiotic Growth

In this study, thermal inactivation and centrifugation were used to inactivate digestive enzymes and separate bioaccessible from non-bioaccessible fraction, respectively, as a simple and non-toxic approach for probiotic bacteria [27]. Nevertheless, it has the disadvantage of not inactivating bile salts, which remain in the pellet (non-bioaccessible fraction) and may affect probiotic growth. Therefore, we also investigated the influence of the digestion blank on the growth of probiotic bacteria in order to isolate the effects of the digestive process.

The digestion blank had a negative impact on the growth of the prebiotic strains, particularly *L. paracasei* subs. *paracasei*. After 24 h of incubation of the digestion blank with *L. paracasei* subs. *paracasei*, there was a significant decrease in growth compared to the negative control. However, after 48 h of incubation, *L. paracasei* subs. *paracasei* appears to have adapted to the digestion blank. Although probiotics are known to survive GID, the stressful conditions of the gastrointestinal tract (bile salts, pH fluctuations, oxygen levels), can compromise their growth, viability and functional properties [28].

**Fig. 1:**
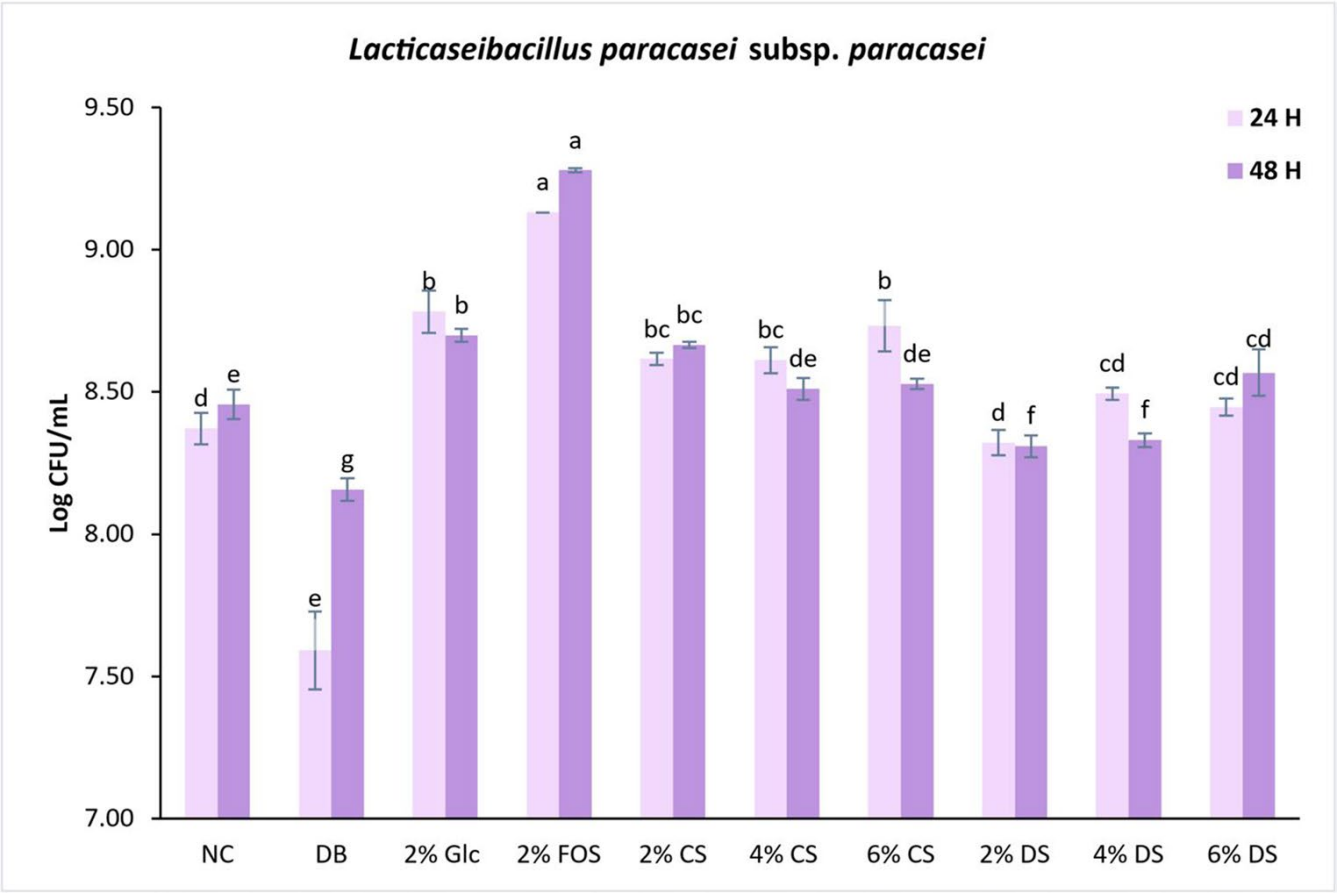
Effect of control and digested coffee silverskin on microbial population of *Lacticaseibacillus paracasei* subs. *paracasei* (24 and 48 h). Values are means ± S.D., *n* = 3. Different letters indicate statistically significant differences (*p* < 0.05) within the same incubation time (24–48 h). *NC*, negative control; *DB*, digested blank; Glc, glucose; *FOS*, fructooligosaccharides; *DS*, digested silverskin;* CS*, control silverskin

**Fig. 2:**
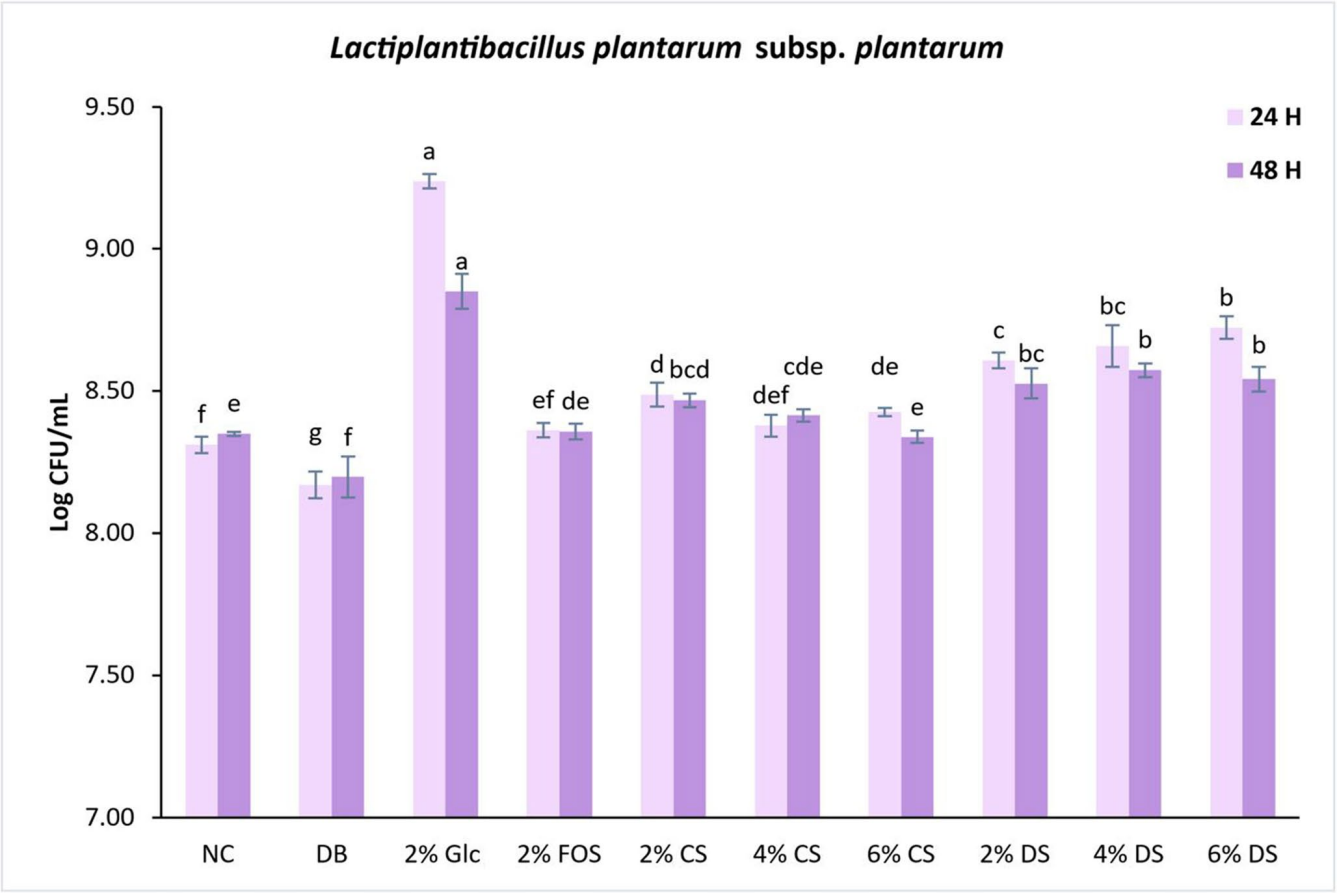
Effect of control and digested coffee silverskin on microbial population of *Lactiplantibacillus plantarum* subs. *plantarum* (24 and 48 h). Values are means ± S.D., *n* = 3. Different letters indicate statistically significant differences (*p* < 0.05) within the same incubation time (24–48 h). NC, negative control; *DB*, digested blank; Glc, glucose; *FOS*, fructooligosaccharides; *DS*, digested silverskin; *CS*, control silverskin

CS and DS promoted the growth of *L. paracasei* subs. *paracasei* (Fig. 1) and *L. plantarum* subs. *plantarum* (Fig. 2) at different levels, suggesting the viability of silverskin as a prebiotic substrate. For *L. paracasei* subs. *paracasei*, after 24 h of incubation, CS at 2 and 6% concentration, significantly enhanced bacterial growth compared to DS (2 and 6%). This difference may be attributed to the sensitivity of *L. paracasei* subs. *paracasei* to the bile salts present in DS, which can interfere with the viability and metabolism of the bacteria [28]. Following 24 h of incubation, DS (4 and 6%) resulted in an insignificant increase in the growth of *L. paracasei* subs. *paracasei* relative to the negative control. All CS concentrations stimulated significantly higher growth of *L. paracasei* subs. *paracasei* than the negative control.

An opposite effect was observed for *L. plantarum* subs. *plantarum* in comparison to *L. paracasei* subs. *paracasei*. After 24 h of incubation, DS (2, 4, and 6%) significantly enhanced the growth of *L. plantarum* subs. *plantarum* compared to CS (2, 4, and 6%). These results suggest that *in vitro* GID may have positively impacted the prebiotic properties of silverskin. This hypothesis will be explored further in the analysis of organic acids. CS (2 and 6%) and DS (2, 4, 6%) significantly boosted bacterial growth than the negative control and FOS. The latter, although an established prebiotic, promoted the growth of *L. plantarum* subs. *plantarum* to a level similar to the negative control, suggesting the strain’s limited ability to utilize this substrate.

These results corroborate previous studies by Machado et al. [12] and Ratnadewi et al. [13], which showed that freeze-dried silverskin extract and silverskin xylooligosaccharides promoted the proliferation of *L. paracasei* subs. *paracasei* and *L. casei*, respectively. Future research should consider dialysis or ultrafiltration (with a larger cut-off size than digestive enzymes and bile salts) to ensure that components of digestion do not adversely affect the growth of probiotic bacteria.

#### pH

**Fig. 3:**
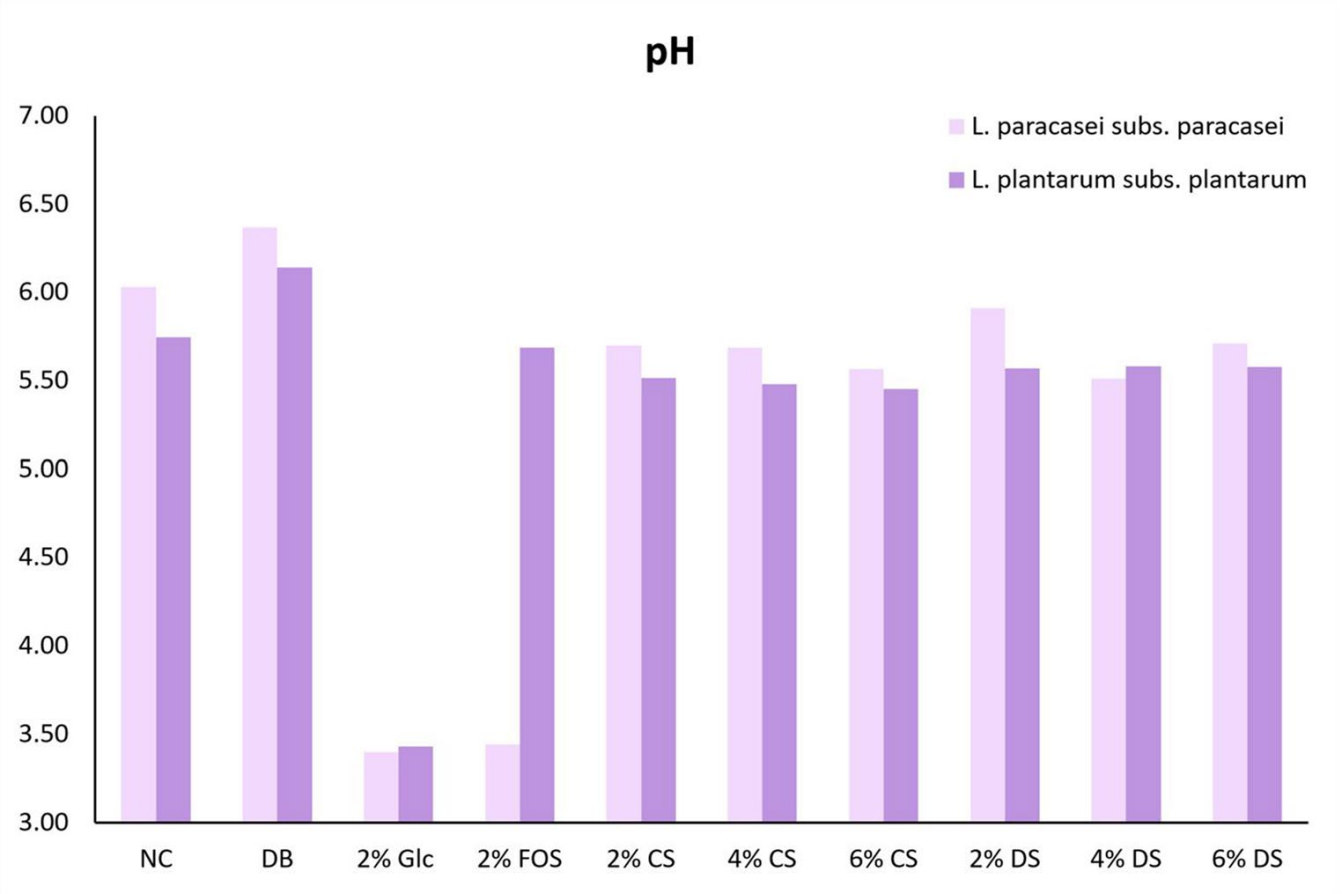
pH of the culture media after 48 h of incubation of *Lacticaseibacillus paracasei* subs. *paracasei* and *Lactiplantibacillus plantarum* subs. *plantarum* with control and digested coffee silverskin. *NC*, negative control; *DB*, digested blank; Glc, glucose; *FOS*, fructooligosaccharides; *DS*, digested silverskin; *CS*, control silverskin

The growth of probiotic strains results in the production of organic acids, leading to a decrease in pH in the surrounding environment. Thus, pH is an important indicator of the degree of fermentation. As shown in Fig. 3, the pH of the CS and DS decreased after 48 h of incubation with the probiotic strains compared to the negative control. This decline in pH shows that the probiotic strains were able to use the silverskin to produce organic acids. The pH variation was more pronounced when the silverskin was incubated with *L. paracasei* subs. *paracasei* than with *L. plantarum* subs. *plantarum*.

#### Organic Acids

The organic acid content shown in Table 2 was analysed after 48 h of incubation of the substrates with *L. paracasei* subs. *paracasei* and *L. plantarum* subs. *plantarum*. Neither strain produce detectable levels of butyric acid or propionic acid when incubated with different substrates (glucose, FOS, CS and DS).

**Table 2:** Organic acids content in cell free supernatants from control and digested coffee silverskin fermented with *Lactiplantibacillus Plantarum* subs. *Plantarum* and *Lacticaseibacillus Paracasei* subs. *Paracasei* (48 h)

	L. plantarum subs. plantarum		L. paracasei subs. paracasei	
Condition	Acetic acid	Lactic acid	Acetic acid	Lactic acid
NC	56.19 ± 0.05ᵃᵇ	n.d	41.20 ± 0.12ᶜᵈᵉ	n.d
DB	37.76 ± 4.56ᵃ	n.d	44.94 ± 0.80ᵉᶠᵍ	n.d
2% FOS	59.81 ± 4.87ᵇ	n.d	35.84 ± 5.40ᵇ	258.09 ± 4.22ᵃ
2% Glc	29.24 ± 8.26ᵃ	233.70 ± 2.40	28.74 ± 4.52ᵃ	259.95 ± 8.24ᵃ
2% DS	137.19 ± 1.00ᵈ	n.d	42.17 ± 0.00ᶜᵈᵉᶠ	n.d
4% DS	90.28 ± 4.06ᶜ	n.d	48.40 ± 1.47ᶠᵍ	n.d
6% DS	127.77 ± 2.16ᵈ	n.d	48.50 ± 0.27ᶠᵍ	n.d
2% CS	335.85 ± 10.06ᵍ	n.d	38.26 ± 0.34ᵇᶜ	n.d
4% CS	290.38 ± 12.88ᶠ	n.d	45.99 ± 1.85ᵉᶠ	n.d
6% CS	250.33 ± 8.90ᵉ	n.d	50.56 ± 0.32ᵍ	n.d

Values are expressed as mM cell free supernatants. Results represent the average of 3 independent experiments ± SD. In each column, different superscript letters represent significant differences between samples (*p* < 0.05). *NC*, negative control; *DB*, digested blank; Glc, glucose; *FOS*, fructooligosaccharides; *DS*, digested silverskin; *CS*, control silverskin

*L. paracasei* subs. *paracasei* cultivated with silverskin (4% DS, 6% DS and 6% CS) led to a significantly higher concentration of acetic acid compared to negative control, glucose and FOS. Nonetheless, *L. paracasei* subs. *paracasei* cultivated with FOS and glucose cultivated led to the production of significant amounts of lactic acid. This organic acid was undetected in the conditions with DS and CS for both strains. Nonetheless, Ratnadewi et al. [13] observed lactic acid production when *L. casei* was cultivated with 20% and 30% silverskin xylooligosaccharides.

*L. plantarum* subs. *plantarum* cultivated with silverskin resulted in high concentrations of acetic acid, especially with CS, indicating enhanced fermentative activity. It should be noted that previous probiotic growth results revealed that *L. plantarum* subs. *plantarum* cultivated with DS exhibited higher growth than that observed with CS. *L. plantarum* subs. *plantarum* may have quickly metabolised the available substrates in CS, with growth declining after 24 h due to the acetic acid accumulation, which, in high concentrations, acts as a fermentation inhibitor [29].

*L. plantarum* subs. *plantarum* and *L. paracasei* subs. *paracasei* are facultative heterofermentative species possessing a diversity of genes related to carbohydrate metabolism. Under normal conditions, these strains metabolise glucose mainly via the Embden-Meyerhof (EMP) homofermentative pathway, producing lactic acid. However, when glucose availability is limited, lactic acid bacteria can use the phosphoketolase (PK) pathway, a heterofermentative metabolism that allows the fermentation of pentoses, such as xylose, resulting in the production of lactic acid and acetic acid in equimolar proportions. This study found xylose as the predominant neutral sugar in silverskin. In addition, a region encoding xylanases and β-xylosidases, enzymes that enable the bacterium the ability to degrade xylan, has been described in *L. plantarum* [30].

In this study, uronic acids were the main carbohydrates detected in silverskin, suggesting the presence of pectic structures. Nazzaro et al. [31] found that *L. plantarum* subsp. *plantarum* can use pectin as a substrate, producing primarily acetic acid and lactic acid, without producing detectable levels of butyric acid.

However, the absence of lactic acid in *L. plantarum* subs. *plantarum* and *L. paracasei* subs. *paracasei* cultures with silverskin suggest that these strains may follow metabolic pathways different from those described in the literature. Alternatively, it is possible that both strains initially produced lactic acid, which was subsequently converted into acetic acid. Guo et al. [32] described the following conversion in *L. brevis* under aerobic conditions, after glucose depletion: lactic acid was oxidised to pyruvate by the reverse action of lactate dehydrogenase; then, pyruvate was converted to acetic acid via pyruvate dehydrogenase or pyruvate oxidase. However, this hypothesis requires further studies, including temporal analyses of metabolites, gene expression profiles and enzyme activity assays, to clarify whether the strains tested produce lactic acid and eventually convert it to acetic acid.

In conclusion, silverskin fermentation with *L. plantarum* subs. *plantarum* and L. *paracasei* subs. *paracasei* resulted exclusively in the production acetic acid, with the former producing higher amounts. Acetic acid is a short-chain fatty acid that helps regulate appetite and body weight. This organic acid can cross the blood-brain barrier and regulate the activity of orexigenic (appetite-promoting) and anorexigenic (appetite-suppressing) neurons in the hypothalamus. At the same time, acetic acid activates GPR43 in adipose tissue, which promotes the production of leptin [33].

#### Antioxidant Activity

Lactic acid bacteria exhibit several probiotic properties, including the enhancement of host antioxidant capacity [34]. Cell free extracts of lactic acid bacteria have been shown to exhibit antioxidant properties by scavenging free radicals, preventing the oxidation of ascorbic and linoleic acids, and promoting the production of antioxidant enzymes [31, 34] Nazzaro et al. [31] found that in the presence of inulin and pectin, *L. plantarum* subs. *plantarum* boosted antioxidant activity (DPPH) 10-fold compared to glucose. DPPH and FRAP assays were used to measure the* in vitro* antioxidant activity of the cell free suspensions obtained after 48 h of incubation of the probiotic strains with different substrates (Table 3).

**Table 3:** *In vitro* antioxidant activity of cell free supernatants from control and digested coffee silverskin fermented with *Lactiplantibacillus Plantarum* subs. *Plantarum* and *Lacticaseibacillus Paracasei* subs. *Paracasei* (48 h), evaluated by DPPH and FRAP assays

	FRAP (FSE)		DPPH^•^-SA (TE)	
Condition	*L. paracasei *subs. *paracasei*	*L. plantarum* subs. *plantarum*	*L. paracasei* subs. *paracasei*	*L. plantarum* subs. *plantarum*
NC	839.55 ± 35.50 ^d^	1033.48 ± 57.56 ^c^	59.00 ± 6.01 ^e^	104.56 ± 5.85 ^c^
DB	260.76 ± 11.44 ^e^	310.76 ± 32.88 ^d^	54.00 ± 0.00 ^e^	102.33 ± 6.67 ^c^
Glc	956.21 ± 36.74 ^d^	913.79 ± 36.46 ^c^	100.67 ± 6.67 ^d^	76.22 ± 3.85 ^e^
FOS	910.76 ± 34.72 ^d^	341.08 ± 3.29 ^d^	108.44 ± 1.93 ^d^	97.57 ± 0.00 ^d^
2% DS	2365.30 ± 143.91 ^b^	1507.73 ± 117.92 ^b^	207.89 ± 23.65 ^b^	177.34 ± 7.64 ^b^
4% DS	3436.51 ± 179.90 ^a^	2251.67 ± 160.47 ^a^	255.66 ± 20.82 ^a^	237.89 ± 19.17 ^a^
6% DS	3427.42 ± 93.64 ^a^	2409.24 ± 20.50 ^a^	274.55 ± 3.85 ^a^	287.89 ± 11.70 ^a^
2% CS	2009.24 ± 64.66 ^c^	1119.85 ± 37.02 ^c^	159.00 ± 9.28 ^c^	209.83 ± 0.84 ^a b^
4% CS	2289.55 ± 122.73 ^b c^	1131.97 ± 105.37 ^c^	187.89 ± 13.88 ^b c^	197.33 ± 6.01 ^b^
6% CS	2363.79 ± 116.80 ^b^	1630.45 ± 94.91 ^b^	194.55 ± 6.94 ^b^	254.00 ± 6.01 ^a^

Values are expressed as µmol/L (FRAP) and mg/L (DPPH) cell free supernatants. Results represent the average of 3 independent experiments ± SD. In each column, different superscript letters represent significant differences between samples (*p* < 0.05). FRAP, ferric reducing antioxidant power; FSE, ferrous sulphate equivalents; DPPH•-SA, 2,2 diphenyl-1picrylhydrazyl radical scavenging activity; TE, Trolox equivalents; NC, negative control; DB, digested blank; Glc, glucose; FOS, fructooligosaccharides; DS, digested silverskin; CS, control silverskin

All the suspensions analysed showed the ability to scavenge free radicals. Both CS and DS combined with the probiotic strains exhibited significantly higher antioxidant activity than the negative, positive and prebiotic controls. This result suggests that the antioxidant capacity of the combination of silverskin and probiotic strain was substantially higher than the individual effects of the probiotic strain. In other words, the presence of bioactive compounds in the CS and DS may have had an additive or even synergistic effect on antioxidant capacity.

The radical scavenging capacity correlated positively with increasing concentrations of CS and DS. The combination of DS (6%) with *L. plantarum* subs. *plantarum* and *L. paracasei* subs. *paracasei* increased antioxidant activity (FRAP and DPPH) by over 2-fold and 4-fold, respectively, compared to the negative control.

Overall, the DS showed higher antioxidant capacity compared to the CS. This result is noteworthy considering that the DS exhibited lower concentrations of caffeine and 5-CQA, as well as lower fermentative activity, in comparison to the CS. One possible explanation is that *in vitro* GID has induced structural changes in the silverskin matrix, increasing the bioaccessibility of certain compounds during probiotic fermentation and thereby enhancing the observed antioxidant activity. Castaldo et al. [15] also reported a significant increase in the antioxidant activity of a silverskin extract after the colonic stage.

Thus, combining probiotic strains with substrates such as silverskin can help reduce the accumulation of free radicals in host tissues and consequently prevent or control diseases associated with oxidative stress such as diabetes, obesity, Alzheimer’s disease, amyotrophic lateral sclerosis, and Parkinson’s disease [35].

## Conclusions

This study provides novel insights into the influence of *in vitro* GID on the chemical composition and potential prebiotic effect of coffee silverskin. After digestion, the carbohydrate fraction exhibited digestive stability, whereas the bioactive chemicals, caffeine and 5-CQA, demonstrated reduced stability, with possible partial release in the bioaccessible fraction. The non-bioaccessible fractions (CS and DS) stimulated the growth of *L. plantarum* subs. *plantarum* and *L. paracasei* subs. *paracasei*, enhanced acetic acid production and consequently decreased pH levels. Nevertheless, the digestion blank exhibited an inhibitory effect, especially on *L. paracasei* subs. *paracasei*, emphasising the importance of methodologies that minimize the influence of residual gastrointestinal fluids. The analysis of the cell free supernatant from the incubations showed antioxidant activity for all the substrates, with a more significant effect observed in the DS. These findings reveal silverskin as a promising and sustainable functional ingredient with applicability in the food and pharmaceutical context.

Despite the promising results, further studies are required to elucidate the metabolic pathways involved in the microbial degradation of silverskin, as well as to apply more realistic intestinal absorption techniques such as dialysis, which can minimise analytical interferences. Moreover, it is imperative to corroborate these results in experiments utilizing human gut microbiota and a wider range of probiotic strains, in order to confirm the functional potential of silverskin.

## Supplementary Information

Below is the link to the electronic supplementary material.

ESM 1 (DOCX 24.3 KB)

## Data Availability

No datasets were generated or analysed during the current study.
