# Supplementary material for: Impact of In vitro Gastrointestinal Digestion on the Chemical Composition and Prebiotic Potential of Coffee Silverskin
Source: Plant Foods Hum Nutr. 2025 Sep 5;80(3):154. doi: 10.1007/s11130-025-01390-z (PMC12413342; doi:10.1007/s11130-025-01390-z)
Supplement: Supplementary file 1 — (DOCX 24.3 KB) [file 11130_2025_1390_MOESM1_ESM.docx]

**Supplementary Section**

**2. Material and methods**

- 1. **Chemicals and analytical standards**

For *in vitro* gastrointestinal digestion, bovine bile salts (B-8631), salivary amylase (A1031), pepsin (P70120), and pancreatin (P7545) were purchased from Sigma Aldrich (Missouri, USA). For carbohydrates determination, sulfuric acid, galacturonic acid, and 3-phenylphenol, were obtained from Merck (Darmstadt, Germany). For quantification of bioactive compounds, analytical standards of caffeine and 3-, 4-, and 5-caffeoylquinic acids were acquired from Sigma-Aldrich (Missouri, USA), and glacial acetic acid from VWR Chemicals (Fontenay-sou-Bois, France). For screening prebiotic effect were used De Man, Rogosa and Sharpe (MRS) broth devoid of glucose, obtained from Liofilchem (Téramo, Italy), and MRS agar with glucose from Scharlab (Barcelona, Spain). Fructooligosaccharides from chicory (FOS), Folin–Ciocalteu reagent, sodium nitrite, ferrous sulfate heptahydrate, epicatechin, Trolox, DPPH• (2,2-diphenyl-1-picrylhydrazyl radical), aluminium chloride, ferric chloride, TPTZ (2,4,6 tripyridyl-S-triazine) sodium acetate, standards of organic acids (lactic, acetic, butyric, propionic acids) from Sigma-Aldrich (Missouri, USA), and sulfuric acid from Merck (Darmstadt, Germany) were also used.

- 1. **Sample**

The coffee silverskin (a 33% *Coffea arabica* and 67% *Coffea canephora* blend) was supplied by the coffee importer and roaster JMV—José Maria Vieira, S.A. (Portugal). After reception, the sample was ground to a fine powder (< 500 µm) using a Thermomix® TM5 (VORWERK, Portugal), vacuum-packed, and stored at room temperature until further analysis.

**2.3 *In vitro* gastrointestinal digestion**

Coffee silverskin (2 g) was submitted to an *in vitro* gastrointestinal digestion according to INFOGEST protocol [1], performed (in duplicate) in three subsequent phases: the oral, gastric and intestinal digestion. Afterwards, to stop small intestinal enzymatic activity, the digested sample was submitted to heat shock at 100 °C for 5 min and then placed on ice. The sample was centrifuged (4 °C, 6000 rpm, 10 min) to separate the bioaccessible fraction (supernatant), absorbed in the small intestine, from the non-bioaccessible fraction (pellet), which proceeds to the colon. A total of 10% of the supernatant was added to the pellet to mimic the fraction that is not readily absorbed after digestion [2]. This combined fractions (pellet + 10 % supernatant), referred to as digested silverskin (DS), was then freeze-dried for further analysis. Additionally, to better distinguish the effects of gastrointestinal digestion on the compounds’ release from those caused by the aqueous medium alone, an aqueous extraction (37 °C for 4 h and 2 min; mimicking the INFOGEST conditions, but without enzymes and reagents) was performed (in duplicate) on coffee silverskin. The resulting pellet combined with 10% of its supernatant was designated as the control silverskin (CS). Also, a digestion blank (DB), in which the silverskin was replaced by deionized water, was run in parallel to account for background signals and potential artefacts arising from the digestion reagent and the process itself.

**2.4 Carbohydrate analysis of non-bioaccessible fractions: CS and DS**

The neutral monosaccharides were analysed as alditol acetates by gas chromatography coupled flame ionization detection (GC-FID) according to Coimbra et al. [3]. For this purpose, 2–3 mg of silverskin (control and digested) were submitted to a pre-hydrolysis with 72% sulfuric acid /w/w) during 3 hours, then hydrolysed with 1 M sulfuric acid at 100 °C for 2.5 h and analysed as alditol acetates using GC-FID. The quantification of uronic acids was performed using a modified colorimetric method with 3-phenylphenol, and the results were expressed as galacturonic acid equivalents. Total carbohydrate content was calculated as the sum of neutral sugars and uronic acids.

**2.7 Quantification of bioactive compounds of non-bioaccessible fractions: CS and DS**

Briefly, 20 mg of sample (CS and DS) were mixed with 900 µL of a hydroethanolic solvent (1:1) and shake at room temperature in a Multi Reax (2000 rpm, 60 min, Heidolph, Schwabach, Germany). Following centrifugation (13000 rpm, 10 min, Biofuge pico Heraeus, Hanau, Germany) the supernatant was collected, and the residue was subjected to a second extraction with 900 µL of a hydroethanolic solvent (1:1) for 30 min. Supernatants were then combined and analysed by HPLC-DAD following the protocol outlined by Machado et al. [4].

Extracts were analysed using a HPLC system (Jasco, Tokyo, Japan). The separation was performed on a Zorbax-SB-C18 (250 mm × 4.6, 5 μm; Agilent Technologies, California, USA). The column operated at 28 °C, with a sample injection volume of 20 µL. The mobile phase was a binary gradient using 0.1% aqueous acetic acid (A) and methanol (B), progressing from 5% to 60% B over 65 min, with a flow rate of 1.1 mL/min. Peak areas were recorded at 274 and 320 nm for caffeine and caffeoylquinic acids (3-, 4-, 5-CQA), respectively. The compounds were identified by comparison of the retention time as well as UV absorption spectra with authentic standards analysed simultaneously.

**2.6 Screening prebiotic effect**

**2.6.1 Probiotic strains and** **inoculum preparation**

The strains *Lacticaseibacillus paracasei* subsp. *paracasei* ATCC® BAA-52™ and *Lactiplantibacillus plantarum* subsp*. plantarum* NCTC ® 13644 (isolated from pickled cabbage) were kept at -20 °C in saline solution containing 0.85% (w/v) NaCl and 20% glycerol. For inoculum preparation, the strain was cultured on MRS agar and maintained at 37 °C for 48 hours. Then, isolated colonies were suspended in saline solution until reaching a turbidity of 0.5 McFarland.

**2.6.2 Evaluation of probiotic growth**

MRS broth was supplemented with non-bioaccessible fractions (CS and DS) at 2%, 4%, and 6% (w/v), all previously autoclaved for sterilisation. In addition, MRS broth was supplemented with 1% (w/v) digestion blank (DB) to evaluate whether digestive fluids and enzymes affect probiotic growth. MRS broth was supplemented with 2% (w/v) of glucose (Glc) as a positive control and 2% (w/v) of fructooligosaccharides (FOS) as a prebiotic. The MRS broths supplemented with Glc and FOS were sterilized by filtration via polyethersulphone membranes with a pore size of 0.22 µm. Simple MRS broth was used as a negative control (NC).

For the evaluation of probiotic growth, non-supplemented or supplemented MRS broth was inoculated (5%, v/v) and incubated at 37 °C for 48 h. After incubation, tenfold serial dilutions were prepared and plated on MRS agar using the single plate-serial dilution spotting (SP-SDS) method [5]. The plates were incubated at 37 °C for 48 h. The colony-forming units were counted, and a logarithmic transformation was applied to the obtained values.

**2.6.3 Measurement of pH**

The pH of each sample was determined at the end of fermentation using a pH-meter (Five Easy Plus PH F20, Mettler Toledo, Greifensee, Switzerland) and consistent values were obtained across measurements.

**2.6.4 *In vitro* determination of antioxidant activity of cell free supernatants**

After 48h of incubation and CFU counts, samples were centrifuged (4 °C, 6 000 rpm, 10 min). The supernatants were filtered using a 0.22 μm syringe filter and named cell free supernatants. These supernatants were used to determine *in vitro* antioxidant activity and organic acid content.

**2.6.4.1 Scavenging of DPPH free radicals**

The radical scavenging capacity of the cell free supernatants was determined according to Costa et al. [6] with minor modifications. The DPPH• was dissolved in ethanol in a 0.06 mM solution. Then, 270 μL DPPH• solution was mixed with 30 μL of sample supernatant or an appropriate dilution (if the initial absorbance was below the required threshold due to excessive DPPH• reduction). The absorbance was measured at 525 nm, in the darkness, every 2 min for 40 min to observe the reaction kinetics using a microplate reader (Gen 5, Synergy HT, BioTek, Vermont, USA). The results were calculated on the basis of the calibration curve of trolox (5.62 - 75.87 mg/L, R^2^ = 0.9993) and were expressed as mg trolox equivalents (TE) per L of cell free supernatant.

**2.6.4.2 Ferric Reducing Antioxidant Power (FRAP) assay**

FRAP assay was carried out according to Benzie & Strain [7] with minor modifications. To prepare the FRAP reagent, 10 mL of acetate buffer (300 mM; pH 3.6) were mixed with 1 mL of TPTZ solution (10 mM in 40 mM HCl), and 1 mL of ferric chloride solution (20 mM). Then, 265 μL of freshly prepared FRAP reagent were mixed with 35 μL of sample supernatant or an appropriate dilution (if the initial absorbance was above the required threshold due to excessive Fe³⁺-TPTZ reduction). The mixture was kept at 37 °C in darkness for 30 min. The reduction of Fe³⁺-TPTZ to the blue-colored Fe²⁺-TPTZ complex was evaluated at 595 nm in a microplate reader (Gen 5, Synergy HT, BioTek, Vermont, USA). Ferrous sulphate was employed to establish the calibration curve (50 to 600 μmol/L; R^2^ = 0.9993) and results were reported as µmol/L of cell free supernatant.

**2.6.5 Analysis of organic acids**

The concentrations of lactic, acetic, propionic, and butyric acids produced by *L. paracasei subs. paracasei* and *L. plantarum* subsp. *plantarum* at the 48 hours of fermentation were determined in accordance with the method suggested by Gullón et al. [8] with minor modifications. Cell free supernatants were analysed using a HPLC integrated system from Agilent (Waldbronn, Germany) comprising a binary pump, degasser, autosampler, column heater, and diode-array detector (Infinity 1260 II, 210 nm). The analytes were separated via Rezex™ ROA-Organic Acid H^+^ (8%) column (300 × 7.8 mm, Phenomenex, Torrance, CA, USA) at 50 °C. The injection volume was 50 μL. The HPLC system operated at flow rate 0.5 mL/min in isocratic mode with mobile phase, 5 mM sulfuric acid. Chromatographic peaks were identified by comparing retention times to those of reference standards and quantified by standard’s curve regression formula.

**Statistical analysis**

All measurements were performed in triplicate. Data were tested for normality and homogeneity by Shapiro–Wilk's and Levene's tests. For the carbohydrate composition, statistical differences were determined by Student's t-test. For the remaining assays, statistical differences were assessed by one-way (ANOVA) followed by Tukey’s HSD tests for multiple pairwise comparisons between the means. *p* values < 0.05 were considered significant. The statistical analysis was conducted using IBM SPSS Statistics version 30.0.0.0 (IBM Corp., Armonk, NY, USA).

**References**

1. Brodkorb A, Egger L, Alminger M, Alvito P, Assuncao R, Ballance S, et al. (2019) Infogest static *in vitro* simulation of gastrointestinal food digestion. Nat Protoc 14:991-1014. https://doi.org/10.1038/s41596-018-0119-1

2. Panzella L, Perez-Burillo S, Pastoriza S, Martin MA, Cerruti P, Goya L, et al. (2017) High antioxidant action and prebiotic activity of hydrolyzed spent coffee grounds (HSCG) in a simulated digestion-fermentation model: Toward the development of a novel food supplement. J Agric Food Chem 65:6452-6459. https://doi.org/10.1021/acs.jafc.7b02302

3. Coimbra MA, Delgadillo I, Waldron KW, Selvendran RR (1996) Isolation and analysis of cell wall polymers from olive pulp. In: (Ed) Plant cell wall analysis edn. Springer, Berlin, pp 19-44

4. Machado M, Espirito Santo L, Machado S, Lobo JC, Costa ASG, Oliveira M, et al. (2023) Bioactive potential and chemical composition of coffee by-products: From pulp to silverskin. Foods 12:2354. https://doi.org/10.3390/foods12122354

5. Thomas P, Sekhar AC, Upreti R, Mujawar MM, Pasha SS (2015) Optimization of single plate-serial dilution spotting (SP-SDS) with sample anchoring as an assured method for bacterial and yeast cfu enumeration and single colony isolation from diverse samples. Biotechnol Rep (Amst) 8:45-55. https://doi.org/10.1016/j.btre.2015.08.003

6. Costa ASG, Alves RC, Vinha AF, Barreira SVP, Nunes MA, Cunha LM, et al. (2014) Optimization of antioxidants extraction from coffee silverskin, a roasting by-product, having in view a sustainable process. Ind Crops Prod 53:350-357. https://doi.org/10.1016/j.indcrop.2014.01.006.

7. Benzie IFF, Strain JJ (1996) The ferric reducing ability of plasma (FRAP) as a measure of “antioxidant power”: The FRAP assay. Anal Biochem 239:70-76. https://doi.org/10.1006/abio.1996.0292

8. Gullón B, Gullón P, Sanz Y, Alonso JL, Parajó JC (2011) Prebiotic potential of a refined product containing pectic oligosaccharides. LWT - Food Sci Technol 44:1687-1696. https://doi.org/10.1016/j.lwt.2011.03.006
